# Pathogenic microbes contaminating mobile phones in hospital environment in Northeast India: incidence and antibiotic resistance

**DOI:** 10.1186/s41182-019-0190-5

**Published:** 2019-12-11

**Authors:** Christine Vanlalbiakdiki Sailo, Puja Pandey, Subhajit Mukherjee, Zothan Zami, Ralte Lalremruata, Lalnun Nemi, Nachimuthu Senthil Kumar

**Affiliations:** 1 Department of Biotechnology, Mizoram University, Aizawl, Mizoram 796004 India; 2 Department of Microbiology, Synod Hospital, Durtlang, Aizawl, Mizoram 796025 India; 3 Department of Pathology, Synod Hospital, Durtlang, Aizawl, Mizoram 796025 India

**Keywords:** Mobile phones, Microorganisms, Acinetobacter, Toilet, Healthcare workers, VITEK 2, Mizoram

## Abstract

**Background:**

The present study attempts to identify and determine the pattern of drug susceptibility of the microorganisms present in mobile phones of health care workers (HCWs) and non-HCWs in a hospital environment. Mobile phones of 100 participants including both genders were randomly swabbed from nine different wards/units and the bacterial cultures were characterized using VITEK 2 system.

**Results:**

Forty-seven mobile phones were culture positive and a total of 57 isolates were obtained which consisted of 28 Gram-positive organisms and 29 Gram-negative organisms. The predominating organisms were *Acinetobacter baumannii* and *Staphylococcus hominis*. Among all the isolates from the mobile phones of HCW and non-HCWs, five isolates had ESBL and three isolates had colistin resistance. Incidentally, MRSA was not found on the mobile phones tested. The isolated organisms showed 100% susceptibility to linezolid, daptomycin, vancomycin, imipenem, meropenem, gentamicin, amikacin, ciprofloxacin and tigecycline, while high resistance was shown against benzylpenicillin (75.0%), cefuroxime and cefuroxime axetil (56.5%). Non-HCWs’ mobile phones were more contaminated as compared to HCWs (*P* = 0.001) and irrespective of individuals’ gender or toilet habits, both Gram-positive and Gram-negative organisms were present on the mobile phones.

**Conclusion:**

This study reports for the first time that the mobile phones of non-health care workers harbour more bacterial diversity and are more prone to cause transmission of pathogens. This study can serve to educate the public on personal hand hygiene practices and on maintaining clean mobile phones through antiseptic measures.

## Background

Mobile phones have become one of the most essential accessories of day-to-day life with recent advances in information sharing and social media applications [1]. The mobile phones contaminated with nosocomial pathogens is a potential mechanical vector for transferring pathogens with multidrug-resistant bacteria, especially extended-spectrum beta-lactamase (ESBL) across various wards in hospital settings [2]. During our daily activities, our hands come in contact with many surfaces containing germs and parallelly handle the phone. Over the course of time, these germs gradually accumulate on our phones and thus, phone and hand hygiene play a very crucial role in our health system. Most of the disease-causing bacteria are transferred from person to person through direct contact and fomites [3].

This study was conducted in Mizoram, which is a landlocked state situated in the north-east of India, with unique lifestyle and food habits. The people of this state are known for their regular consumption of boiled and fermented foods and there are strong evidences of the impact of fermented foods on the digestive health such as dysbiosis and well-being [4]. The effects of certain lifestyle and food habits influence the gut microbiota which in turn can inhabit the mobile phones of their owners [5]. This study aims to survey the diversity of culturable microorganisms present on mobile phones which can vary owing to differences in hand flora, diet, occupation, habits and health awareness.

The key questions addressed in this study are as follows: (1) What is the contamination rate of mobile phones in a hospital environment and what are the predominant pathogens present? (2) Which are the most resistant microorganisms and what are their antibiotic resistance profiles? (3) What are the most important factors responsible for mobile contamination? In this study, identification and pattern of drug susceptibility of microorganisms isolated from mobile phones of both health care workers (HCWs) and non-health care workers (non-HCWs) in a hospital environment was carried out.

## Results

Of the total 100 mobile phones swabbed, 47 samples showed positive growth (47%)—from which 57 bacterial isolates were identified (28 Gram-positive organisms and 29 Gram-negative bacilli). Twenty-one mobile phones (44.7%) were culture positive for Gram-positive organisms alone (including single and multiple organisms), while 23 mobile phones (48.9%) were culture positive for Gram-negative bacilli (GNB) alone (including single and multiple organisms) and 3 mobile phones (6.4%) had mixed growth of both Gram-positive and negative organisms (Tables 3 and 4).

### Antimicrobial susceptibility testing (AST)

The AST of Gram-positive cocci (GPC) and GNB from both HCWs and non-HCWs are shown in Tables 3 and 4. Among the GNBs, out of five ESBL isolates (sample code MP-25, MP-26, MP-71, MP-80, MP-92), four were *Enterobacter cloacae* (one patient attendant from the Gynaecology ward, two patient attendants from the Medical ward and one patient from the Orthopaedic ward) and one was *E. coli* (female ward attendant from the Surgical ward). It is noteworthy that in the total three *E. coli* strains isolated, two were non-ESBL (sample code MP-60, MP-82) from non-HCWs belonging to the Gynaecology ward and Surgical ward, while one ESBL strain (sample code MP-80) was from HCW of the Surgical ward. We also identified two strains of *Klebsiella pneumoniae* which were non-ESBL producers (Table 3).

Among the GNB, colistin resistance was shown by three isolates—*E. coli* (HCW, Surgical ward, sample code MP-80), *Moraxella* group and *S. paucimobilis* (non-HCWs, Gynaecology ward, sample codes MP-67 and MP-70). Intermediate drug resistance was shown by the *Moraxella* group against ceftriaxone and ampicillin as well as by *S. paucimobilis* against cefuroxime axitel and cefepime. These organisms were isolated from non-HCWs belonging to the Gynaecology ward. In GPC, intermediate resistance was shown against erythromycin by *S. cohnii* (non-HCW, Medical ward) and *S. xylosus* (HCW, k-ward) and against teicoplanin by *S. hominis* (HCW, ENT ward). Of the 29 GNB isolated, six isolates (*Pantoea* spp., *Oligella ureolytica*, *Klebsiella pneumoniae*, *Aeromonas salmonicida*, and two strains of *Acinetobacter lwoffii*) could not produce AST results when tested by VITEK 2 and therefore are not included in AST.

In this study, we observed that the most resistant GNB was *Enterobacter cloacae* (ESBL) isolated from non-HCW belonging to the Medical ward (sample code MP-26). This isolate showed resistance to 10 antibiotics (55.55%) out of the 18 antibiotics tested. On the contrary, the most susceptible GNB was *Sphingomonas paucimobilis* isolated from a HCW belonging to Microbiology laboratory (sample code MP-4). This isolate showed susceptibility to all the 18 antibiotics tested (100%) (Table 3). Conversely, the most resistant GPC was *S. haemolyticus* isolated from non-HCW belonging to the Medical ward (sample code MP-23). This isolate showed resistance to 10 antibiotics (62.5%) out of the 16 antibiotics tested. The most susceptible (100%) Gram-positive cocci were *S. sciuri* (isolated from HCW, ENT ward, sample code MP-39), *S. warneri* (isolated from non-HCW, ENT ward, sample code MP-31) and *S. hominis* (isolated from non-HCW, Gynae ward, sample code MP-69). These isolates showed susceptibility against all the antibiotics tested (Table 4).

Among the GPC, there were eight isolates of coagulase-negative *Staphylococcus* (CoNS) that had shown resistance to beta-lactams as well as modification of penicillin-binding protein (mecA). These included four strains of *S. haemolyticus* (sample codes MP-23, MP-24, MP-29, MP-35) which were from non-HCWs (Medical ward and ENT ward), three strains of *S. hominis* (sample codes MP-39, MP-47, MP-72) which were from HCWs (ENT ward) and non-HCWs (Gynaecology ward) and one *S. epidermidis* (sample code MP-85) from non-HCW (Surgical ward).

### Single/multiple and mixed growth with respect to wards/units

Eight wards/units had either single/multiple and/or mixed growth of both Gram-positive and Gram-negative organisms. Interestingly, in the Orthopaedic ward, all the isolates obtained were GNBs. Mobile phones swabbed from ENT (72.7%) and Gynaecology (56.2%) wards were more contaminated as compared with other wards/units (Table 1).

**Table 1:** Bacterial growth from mobile phones belonging to different wards/units

Wards/units	No. of samples	No. of growth (%)	Total no.			Organisms isolated
			Isolates	GNB’s	GPC’s/GPB	
Microbiology unit	05	02 (40.00)	02	01	01	1 *Sphingomonas paucimobilis*, 1 *Staphylococcus aureus* (MSSA)
Blood bank unit	04	01 (25.00)	01	00	01	1 *Staphylococcus cohnii*
Pathology unit	09	01 (11.11)	01	00	01	1 *Staphylococcus arlettae*
Medical ward	11	06 (54.54)	07	02	05	3 *Staphylococcus hemolyticus*, 2 *Enterobacter cloacae*, 1 *Staphylococcus cohnii* and 1 *Alloiococcus otitis*
ENT ward	11	08 (72.72)	13	03	10	2 *Staphylococcus warneri*, 2 *Staphylococcus hominis*, *1 Staphylococcus epidermidis*, 2 *Staphylococcus saprophyticus*, 1 *Acinetobacter lwoffii*, 1 *Pantoea* spp., 1 *Kocuria kristinae*, 1 *Acinetobacter hemolyticus*, 1 *Staphylococcus sciuri* and 1 *Staphylococcus hemolyticus*
K ward	04	01 (25.00)	01	00	01	1 *Staphylococcus xylosus*
Gynaecology ward	32	18 (56.25)	20	13	07	4 *Acinetobacter baumanii*, 1 *Enterobacter cloacae*, 4 *Staphylococcus hominis*, 2 *Acinetobacter lwoffii*, 2 *Moraxella group*, 1 *Sphingomonas paucimobilis*, 1 *Staphylococcus aureus*, 1 *Pantoea* spp., 1 *Staphylococcus epidermidis*, 1 *Klebsiella pneumoniae*, 1 *Erysipelothrix rhushiopathiae* and 1 *E.coli*
Surgical ward	15	05 (33.33)	07	05	02	2 *Escheriachia coli*, 1 *Acinetobacter baumanii*, 1 *Staphylococcus epidermidis*, 1 *Klebsiella pneumoniae*, 1 *Enterococcus* spp. and 1 *Oligella ureolytica*
Orthopaedic ward	09	05 (55.55)	05	05	00	2 *Acinetobacter lwoffii*, 1 *Acinetobacter baumanii*, 1 *Enterobacter cloacae* and 1 *Aeromonas salmonicida*

*GNB* Gram-negative bacilli, *GPC* Gram-positive cocci

*K ward* substance abuse ward, *ENT* ear, nose and throat ward

### Bacterial isolates from mobile phones in relation to different parameters

An informed verbal consent was obtained and a set of questionnaire was asked to all the participants pertaining to gender, occupation [HCWs, non-HCWs (patients and patient attendants), their associated hospital wards/units, habit of cleaning their mobile phones and of carrying their phones to toilet (Table 2).

**Table 2:** Relationship between bacterial isolate numbers and different parameters

No. of bacterial isolates						
Factors	No growth	Growth per mobile phone^a^	Total no. of isolates^b^	*p* value	OR	CI
	No. (%)	No. (%)	No. (%)			
Gender						
Male	17 (53.12)	15 (46.88)	19 (33.33)	0.680	1.194	0.515–2.769
Female	38 (55.88)	30 (44.12)	38 (66.67)			
Occupation						
HCW	26 (76.47)	08 (23.53)	10 (17.54)	0.001	4.694	1.848–11.922
Non-HCW	27 (40.90)	39 (59.10)	47 (82.46)			
Mobile cleaning						
Yes	13 (56.52)	10 (43.48)	15 (26.32)	Reference	3.756	1.240–11.373
Never	39 (50.65)	38 (49.35)	42 (73.68)	0.019		
Use of mobile in the toilet						
Never	09 (50.00)	09 (50.00)	11 (19.30)	Reference	1.202	0.471–3.072
Yes	43 (52.44)	39 (47.56)	46 (80.70)	0.700		
Wards/Units						
Surgical ward	10 (66.67)	05 (33.33)	07 (12.28)	Reference		
Pathology unit	08 (88.89)	01 (11.11)	01 (1.75)	0.246	0.250	0.024–2.594
Microbiology unit	03 (60.00)	02 (40.00)	02 (3.51)	0.787	1.333	0.165–10.743
Blood bank unit	03 (75.00)	01 (25.00)	01 (1.75)	0.751	0.667	0.054–8.161
Medical ward	05 (45.45)	06 (54.55)	07 (12.28)	0.284	2.400	0.484–11.891
ENT ward	03 (27.27)	08 (72.73)	13 (22.81)	0.055	5.333	0.968–29.393
K ward	03 (75.00)	01 (25.00)	01 (1.75)	0.751	0.667	0.054–8.161
Gynaecology ward	14 (43.75)	18 (56.25)	20 (35.10)	0.148	2.571	0.714–9.255
Orthopaedic ward	04 (44.44)	05 (55.56)	05 (8.77)	0.290	2.500	0.458–13.649

References represent the variable against which the tested factors were compared

*OR* odds ratio, is a measure of association between an exposure and an outcome; *CI* confidence interval, indicates a measurement precision. Narrow CI indicates high precision; Wide CI indicates low precision; *p* value, the probability of finding the observed results when the null hypothesis is true (indicates significance < 0.05); *ENT* ears, nose and throat; *K ward* substance abuse ward

^a^Growth observed per individual’s mobile phone

^b^Total no. of bacterial isolates from each individual’s mobile phone (includes > 1 organism per mobile phone)

**Fig. 1:**
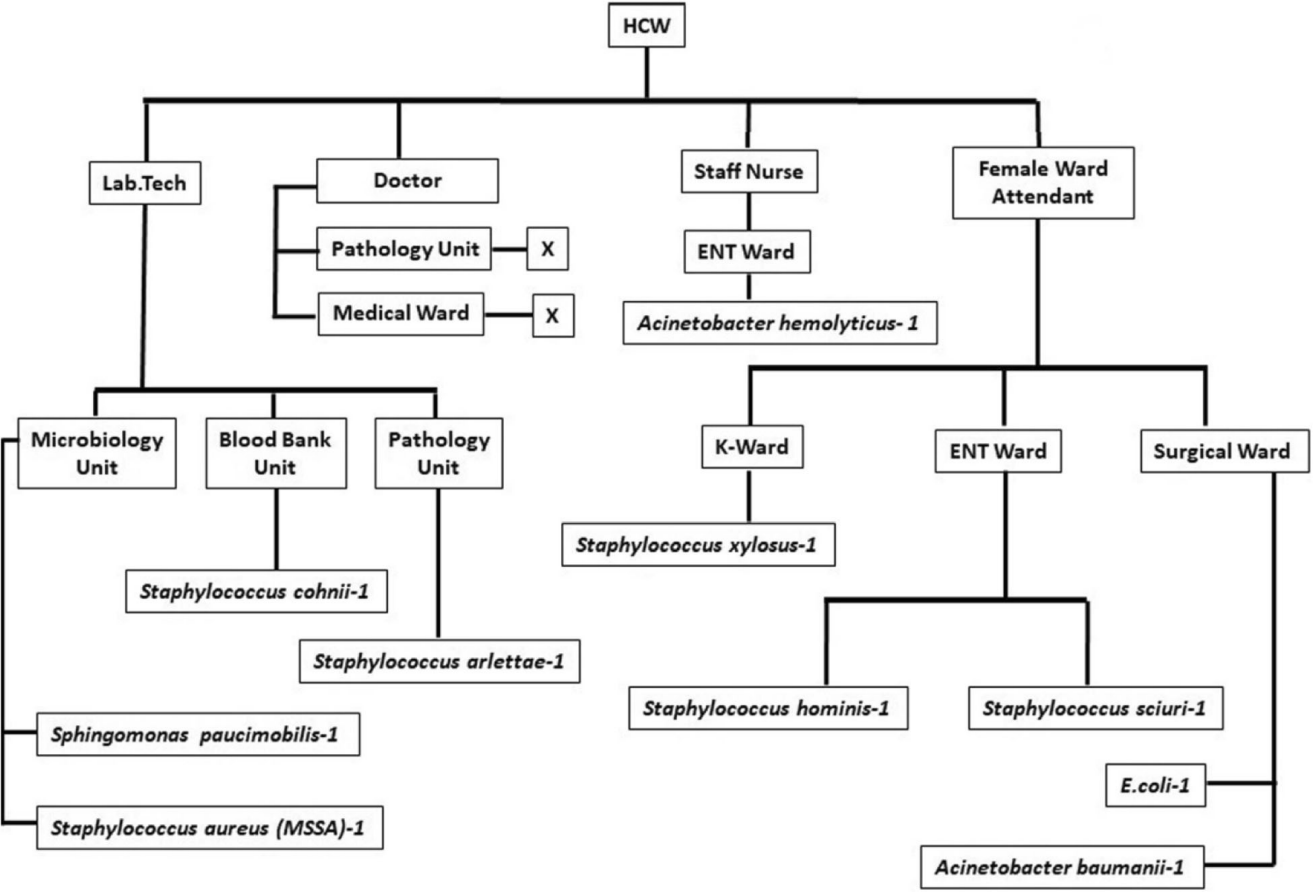
Bacterial isolates obtained from health care workers in a hospital environment. K-ward, substance abuse ward. *X*, Nil microorganisms isolated

**Fig. 2:**
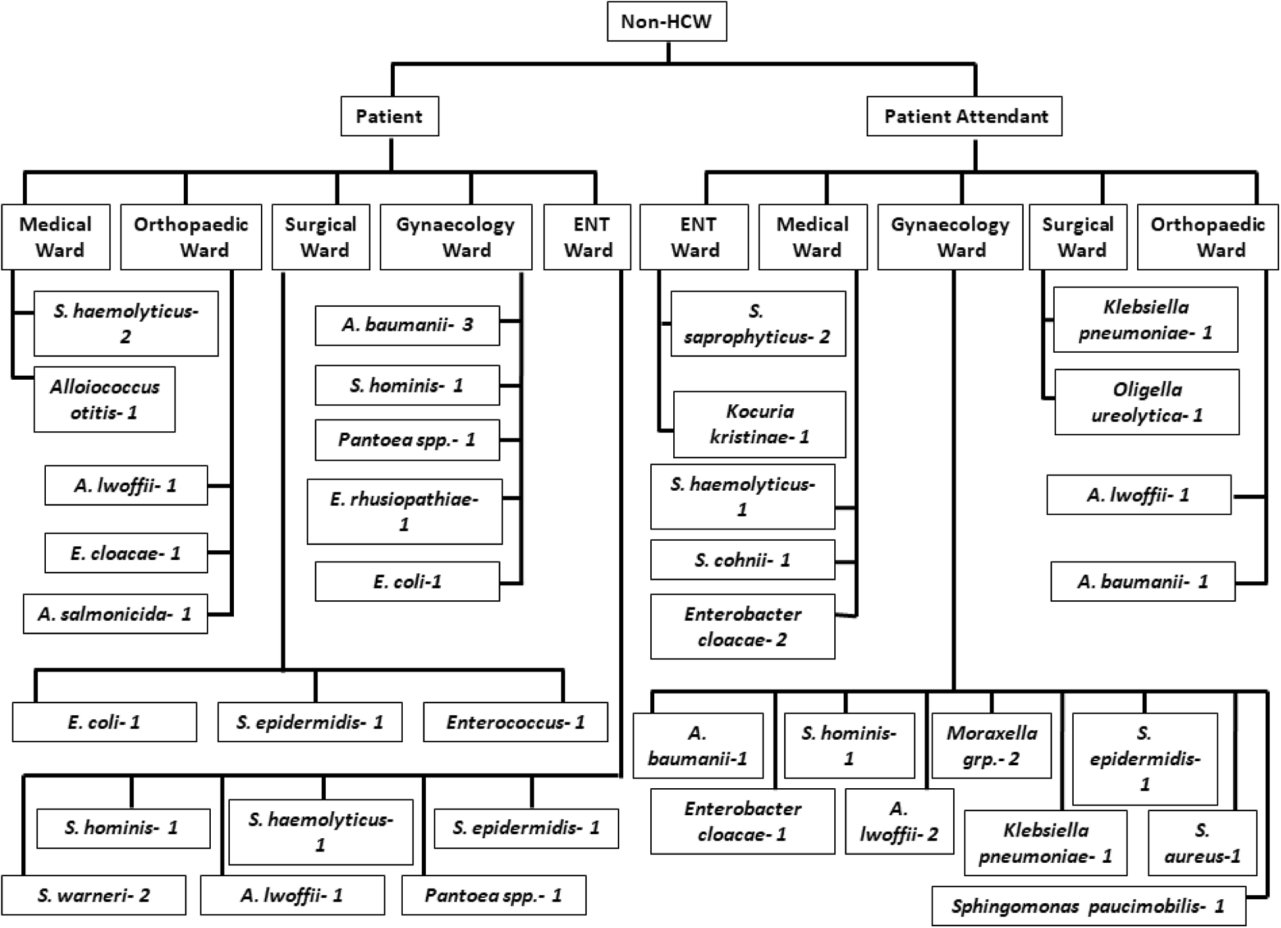
Bacterial isolates obtained from non-health care workers in a hospital environment. 1, 2 and 3 represents the number of organisms isolated

Out of 100 participants, 32 were males and 15 (46.9%) of their mobile phones were culture positive, whereas 68 were females and 30 (44.1%) of their mobile phones were culture positive (*p* value = 0.680; OR = 1.194; CI = 0.515–2.769). With respect to occupation, out of the 100 participants, 66 were non-HCWs and 34 were HCWs. The bacterial contamination rate of mobile phones among the non-HCWs was 59.1% and HCWs was 23.5%. We observed that non-HCWs mobile phones had more contamination (*p* = 0.001; OR = 4.694; CI = 1.848–11.922) as compared to HCWs (Table 2). With respect to occupation, the type of organisms isolated revealed that non-HCWs phones were more contaminated with *Acinetobacter baumannii* (*n* = 5) and *Acinetobacter lwoffii* (*n* = 5). On the contrary, there were no predominating organisms in the mobile phones of HCWs (Figs. 1 and 2).

**Fig. 3:**
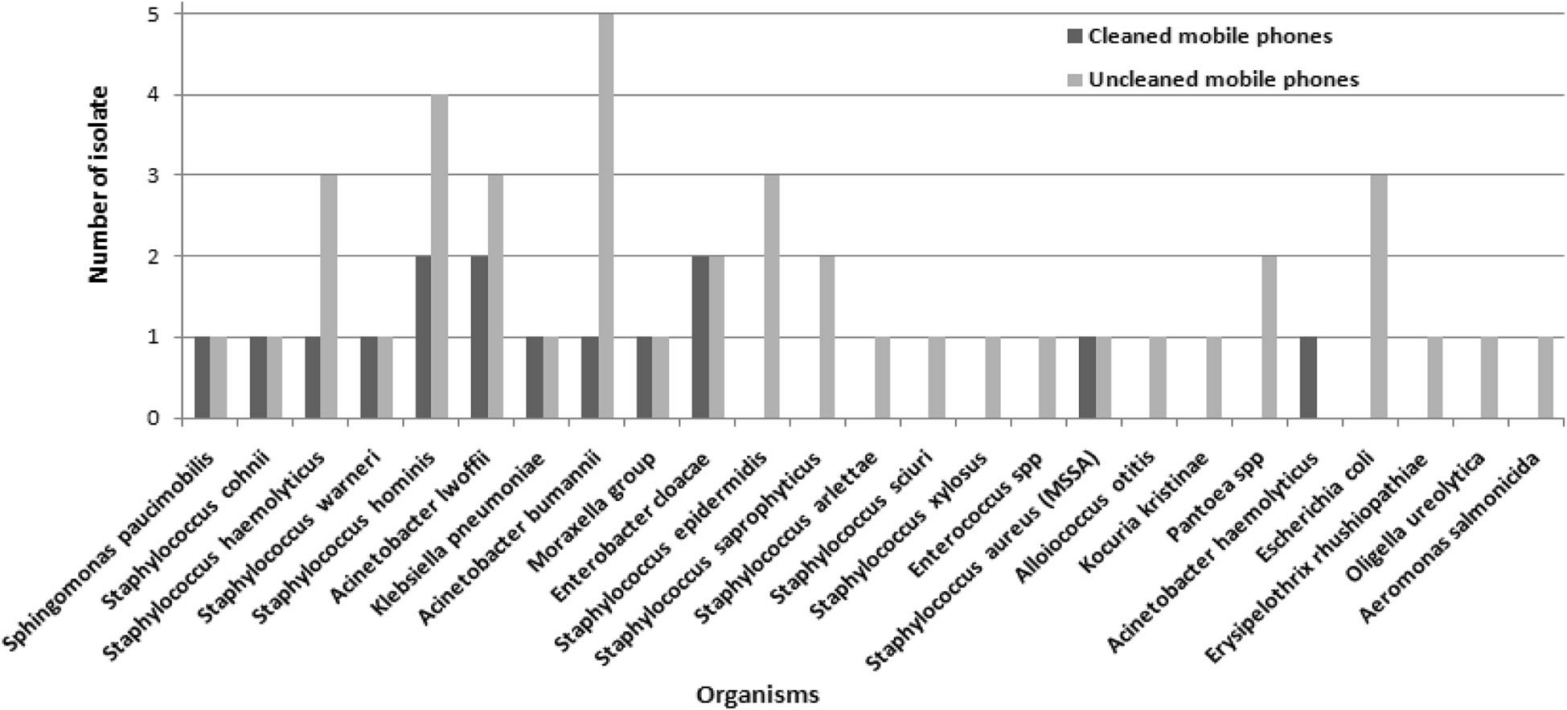
Bacterial isolates isolated from mobile phones that were and were not cleaned. This figure shows that more number of microorganisms are present on uncleaned mobile phones

With respect to the cleanliness of mobile phones, 23 participants had cleaned (without antiseptics) and 77 participants never cleaned their mobile phones (Table 2). Among these 23 participants, 10 samples were culture positive (43.4%) and maximum growth was contributed by *Enterobacter cloacae*, *Acinetobacter lwoffii* and *S. hominis*. The remaining were *Sphingomonas paucimobilis*, *S. cohnii*, *S. haemolyticus*, *S. warneri*, *Acinetobacter baumannii*, *Moraxella* group, *S. aureus* (MSSA) and *A. haemolyticus* (Fig. 3)*.*

From the 77 uncleaned mobile phones, a total of 24 organisms were isolated (31.16%) (Table 2). *Acinetobacter baumannii* was abundant, followed by *S. hominis, S. haemolyticus*, *Acinetobacter lwoffii*, *S. epidermidis* and *E. coli.* Apart from the common organisms that grew both in the cleaned and uncleaned mobile phones, the uncleaned phones had additional growth of organisms such as *Klebsiella pneunoniae*, *S. epidermidis*, *S. saprophyticus*, *S. arlettae*, *S. sciuri*, *S. xylosus*, *Enterococcus* spp., *Alloiococcus otitis*, *Kocuria kristinae*, *Pantoea* spp., *E. coli*, *Erysipelothrix rhusiopathiae*, *Oligella ureolytica* and *Aeromonas salmonicida* (Fig. 3). The contamination rate was significantly higher in the uncleaned phones compared to the cleaned ones (statistical findings from Table 2).

**Fig. 4:**
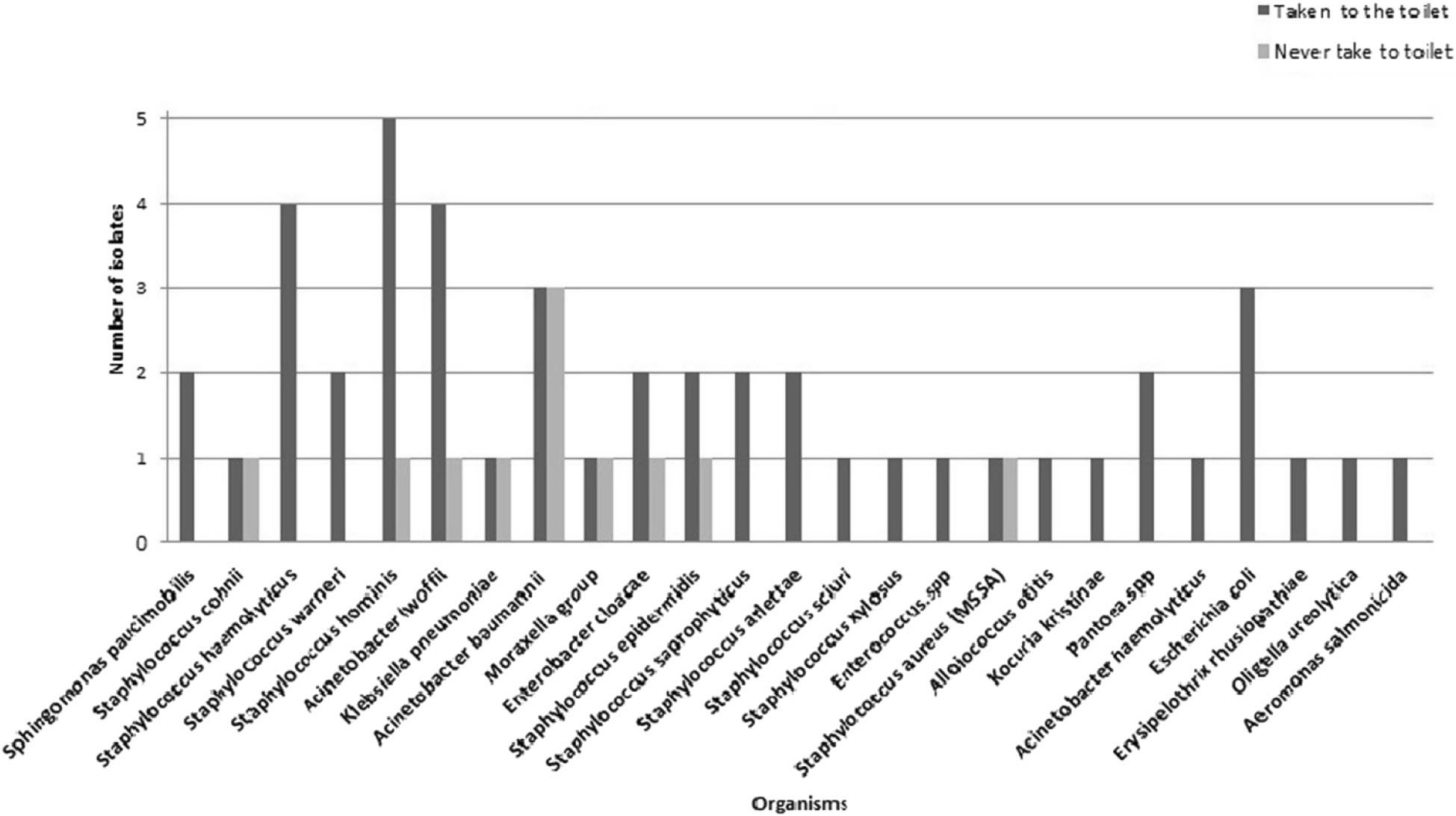
Bacterial isolates from mobile phones that were and were not taken inside the toilet. This figure shows that more number of microorganisms are present on mobile phones that were taken inside the toilet

Eighty-two (82) individuals used to carry their mobile phones inside the toilet, out of which 39 mobile phones (47.5%) were culture positive yielding 46 isolates (Table 2). In total, these isolates contained 25 types of organisms with a dominance of *S. hominis* (Fig. 4). Nine (50%) out of 18 mobile phones that were never carried inside the toilet were culture positive and from them, 11 isolates (containing 9 types of microbes) were identified with a dominance of *Acinetobacter baumannii* (Table 2, Fig. 4). There was no statistical significance in the number of microbial growth between the mobile phones that were and were not carried inside the toilet (*p* = 0.700) (Table 2).

## Discussion

Nowadays, mobile phones are being owned and widely used everywhere by almost every individual irrespective of age, socio-economic status and educational level. It, therefore, has become an easy source of microorganism transmission. In the present study, 47% of the mobile phones were contaminated, whereas earlier studies had shown mobile phone contamination up to 94.5% and 30% [6, 7]. This variation could be attributed to knowledge of hand hygiene and sanitary practices and with rapid dissemination of awareness and precautions. This study proved that the mobile phones harbour both Gram-positive organisms (*n* = 28, i.e., 49.1%) and Gram-negative bacilli (*n* = 29, i.e., 50.9%) irrespective of belonging to HCWs or non-HCWs. Though this figure is not significantly high, more number of GNB was isolated. This is not in agreement with other studies where Gram-positive organisms (80%) were abundant rather than Gram-negative organisms (20%) [8, 9].

For GNB, the antibiotic resistance and susceptibility pattern among microbes isolated from mobile phones were almost similar to other previous studies [10], except for ampicillin (34.78%), piperacillin/tazobactam (4.35%) and cefuroxime axetil (56.52%) (Table 3). This low susceptibility to cefuroxime axetil can be attributed to the fact that it falls under the commercially available second generation of cephalosporins which were once widely used as therapy. Cefuroxime axetil was active against a wide range of organisms and its use decreased later due to the acquisition of resistance which led to the use of broader classes of cephalosporins like third, fourth and fifth generations [11].

**Table 3:**
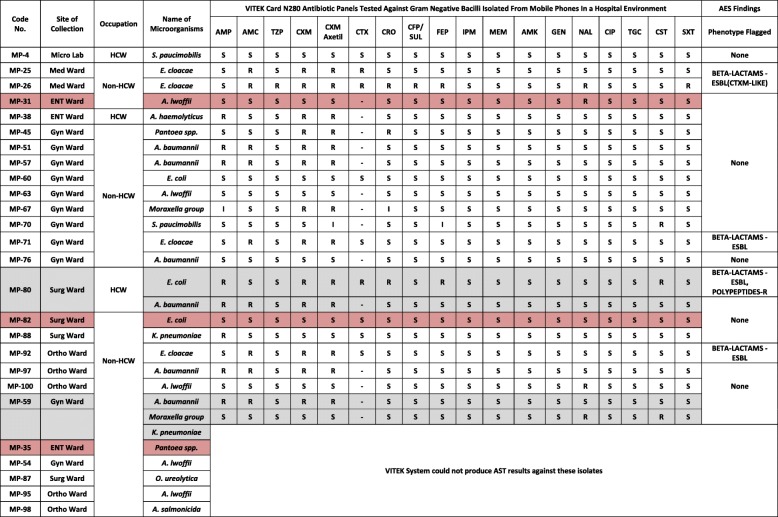
Antibiotic susceptibility and resistance pattern for GNB

*AMP* ampicillin, *AMC* amoxicillin/clavulanic acid, *TZP* piperacillin/tazobactam, *CXM* cefuroxime, *CTX* cefotaxime, *CRO* ceftriaxone, *CFP/SUL* cefoperazone/sulbactam, *FEP* cefepime, *IPM* imipenem, *MEM* meropenem, *AMK* amikacin, *GEN* gentamicin, *NAL* nalidixic acid, *CIP* ciprofloxacin, *TGC* tigecycline, *CST* solistin, *SXT* trimethoprim/sulfamethoxazole, *S* susceptible, *R* resistant

Grey highlight—mobile phones having growth of > 1 organisms of the same Gram reaction

Pink highlight—mobile phones having mixed growth of both Gram-positive and Gram-negative organisms

In concordance with previous studies, GNB had 100% susceptibility to imipenem, meropenem, amikacin and ciprofloxacin [12]. This can be attributed to the fact that the carbapenems such as imipenem and meropenem are reserved drugs which are seldom used, unless needed, and hence, GNB showed good susceptibility (100%) towards those drugs. This is in accordance with other previous studies [13]. It is noteworthy to mention that mobile phones of patient attendants (non-HCWs) and female ward attendants (HCWs) harboured extremely important nosocomial pathogens which were extended spectrum beta-lactamase (ESBL) microorganisms such as *Enterobacter cloacae* and *E. coli*. Such individuals can act as a source of ESBL transmission from patient-to-patient, patient-to-healthcare workers and patient/ward attendants to patients during hospitalization [14, 15]. In our study, of the three isolated *E. coli* strains, two were non-ESBL producers and one was ESBL producer. In addition, two *Klebsiella pneumoniae* strains isolated from patient attendants of surgery and Gynae wards were non-ESBL producer. That *E. coli* and *Klebsiella* can be both ESBL producer and non-ESBL producer is supported by a previous study [16].

Three colistin resistance strains were identified among all the GNB isolates obtained. These are *E.coli* (HCW, Surgical ward), *S. paucimobilis* and *Moraxella* group (non-HCWs, Gynaecology ward). However, only *E. coli* was flagged as polypeptides resistant by VITEK 2 system*.* Since colistin is a last resort drug and the associated resistance gene is transmissible to other drug naïve bacterial species as well as pathogens, carriage of the resistance gene to pathogens harbouring other resistance genes can give rise to super drug-resistant bacteria [17]. In this study, the presence of a colistin-resistant *E. coli* strain on a HCW signifies the alarming chances of it being transmitted across various wards which could eventually lead to a great hazard in clinical practice.

CoNS are well known opportunistic pathogens in hospital settings that result in high-end infections since they have the ability to adhere and invade epithelial cell lines, like HeLa [18] and form biofilms in prosthetic devices ultimately resulting in drug resistance [19, 20]. In this study, overall, 28 Gram-positive isolates were identified of which 22 were contributed by CoNS. It is not clear to us as to what extent these CoNS would have infected patients from this hospital.

As an antibiotic, benzylpenicillin is noted to possess efficacy against a wide variety of infections [10]. In the present study, the GPC had 75% resistance against benzylpenicillin, whereas it was found to be 100% in an earlier study [21]. This pattern of high resistance towards this antibiotic could be due to the rampant utilization of different groups of antibiotics including penicillin group of drugs, not only for empirical treatment but also for non-curative reasons like prophylaxis and metaphylaxis in animal feed stocks which thereby promotes drug resistance [22]. The GPC in our findings showed 100% susceptibility towards linezolid, vancomycin and tigecycline which is in total agreement with previous finding [23]. This could be due to the fact that they fall under second line drugs which are seldom used unless needed and hence showed good susceptibility [12].

In this study, no significant gender association was found between the organisms isolated from mobile phones of both male and female participants. With respect to occupation, mobile phones of non-HCWs (patient and patient attendants) were significantly contaminated (*p* = 0.001) as compared to those of HCWs and were predominantly contaminated with *A. baumannii* and *A. lwoffii*. This can be attributed to the proper hand hygiene awareness and practices among the HCWs resulting in the constant sterilization of hands either by hand-washing or using sanitizer upon touching patients and hospital devices. Hence, hand hygiene is the leading measure for preventing the transmission of antimicrobial resistance and reducing healthcare-associated infections [24].

As anticipated in this study, mobile phones which were never cleaned had more microorganisms (*p* = 0.019) and *A. baumannii*, *S. hominis*, *A. lwoffii*, *S. haemolyticus*, *S. epidermidis and E. coli* dominated other microorganisms (Fig. 3, Table 2). A similar finding was also reported by Koscova et al. [25] where the researchers also concluded that cleaning of mobile phones especially with antibacterial solutions can markedly reduce microbial contamination with an effective range of 36.8 to 100%.

Previous studies have regarded toilets as a source of microbial contamination and a hidden source of microbial transmission of pathogens across various individuals [26]. We found more number of microorganisms on mobile phones that were carried inside the toilet, and among them, *S. hominis* predominated (Fig. 4). Various sub-species of this species had been implicated for nosocomial outbreaks causing bloodstream infections in patients with underlying malignancies [27].

Our sampling was carried out during summer and the genus *Acinetobacter* as such are part of the human skin flora which shows seasonal variability of 53% during summer vs 32% during winter [28, 29]. In this study, GNBs such as *Acinetobacter lowffii*, *Acinetobacter baumannii* and *E. coli* predominated in the mobile phones taken inside the toilet, and interestingly, an equal number of *Acinetobacter baumannii* was also found among those mobile phones that were never taken inside the toilet (Fig. 4). *Acinetobacter baumannii* has been reported to colonize the skin at very low levels and is an emerging multidrug-resistant pathogen in community and hospital environments which recognizes them as the most difficult GNB to control and treat [30–32]. *Acinetobacter* spp. have been reported to colonize up to 75% in hospitalized patients vs 43% of community dwellers and among these community dwellers, *Acinetobacter lowffii* contributed 58% [33]. Other microorganisms such as *S. cohnii*, *K. pneumoniae*, *Moraxella* group and *S. aureus* (MSSA) were also found in equal percentage in both the groups in the present study (Fig. 4).

We also observed that in our study, most of the microorganisms tested against the panel of antibiotics were of susceptible strains, which indicates that these microorganisms might have come from the community through the patients, patient attendants and/or hospital staff. Over time, such microorganisms can acquire resistance and infect patients when the opportunity arises and can even be fatal. Organisms like *S. aureus* (MRSA), CoNS and ESBL are well known for bloodstream infections leading to septicaemia and death [34]. The main drawback of this study apart from sample size is that an equal number of sampling from different sources could not be carried out due to the unavailability of an equal number of patients at the time of sample collection.

## Conclusion

From this study, the most important factor associated with mobile contamination was being unaware of the facts that mobile phones can harbour microorganisms and mobile phones need to be cleaned with antiseptics. The novel finding of our work is that the mobile phones of non-health care workers are more contaminated and are more prone to cause transmission of pathogens. This study can create public awareness regarding the danger of transmission of germs through mobile phones and can educate the people about hand hygiene practices and regular cleaning of mobile phones with antiseptics.

## Materials and methods

### Sample collection

A total of 100 mobile phone swab samples were aseptically collected from a mission hospital using sterile cotton swab in screw-capped polypropylene tubes (HiMedia PW003-1x100NO) moistened with 9% sterile normal saline. The entire surface of the phone including the screen, buttons of keypads, sides and back, mouth and ear piece, volume and lock keys and the cover inside out were thoroughly swabbed [35]. Participants were both HCWs and non-HCWs and were randomly selected as per their convenience at the time of sample collection during the Summer period (March–May 2018). Mobile phone swab samples were collected from nine locations (six wards and three units) within the hospital as follows: 18 samples from three laboratory units (Microbiology—5, Blood bank—4 and Pathology unit—9) and 82 samples from six wards (Surgical ward—15, Medical ward—11, ENT ward—11, K-ward—4, Gynaecology ward—32, Orthopaedic ward—9) (Table 2). Further, the 82 samples were from patient attendants—37, patients—29, female ward attendants—4, staff nurse—11 and ward doctor—1 and the 18 samples were from Lab technicians—17 and laboratory doctor—1.

### Sample processing and identification

All samples were processed at the Microbiology laboratory in the mission hospital, Mizoram, by plating on sheep blood agar plate (HiMedia MP1301), MacConkey agar (HiMedia MH081) and chromogenic media (HiCHROME UTI Agar M1353) using the three-phase streaking pattern and incubating aerobically at 37 °C for 24–36 h. Gram staining (HiMedia K001) was performed on isolated colonies and identified using VITEK 2 system (BioMérieux, Biotechnology Company; France). VITEK identification cards were selected as per the morphology and Gram reaction of the bacteria [36]. This automated system monitors the kinetics of bacterial growth, calculates it using a unique algorithm and follows a Clinical and Laboratory Standards Institute (CLSI) guidelines [37].

### Antibiotic susceptibility testing

For antibiotic susceptibility test (AST), the VITEK 2 system was used as per manufacturer’s instructions. For GNB, N280 antibiotic cards which included a panel of 18 antibiotics were used (Table 3). Etrapenem was not included since it did not produce any results upon testing by VITEK 2. For GPC, P628 antibiotic cards with a panel of 16 antibiotics were used (Table 4). Cefoxitin was used as a surrogate marker for methicillin-resistant *S. aureus*. Rifampicin did not produce any results upon testing and thus excluded.

**Table 4:**
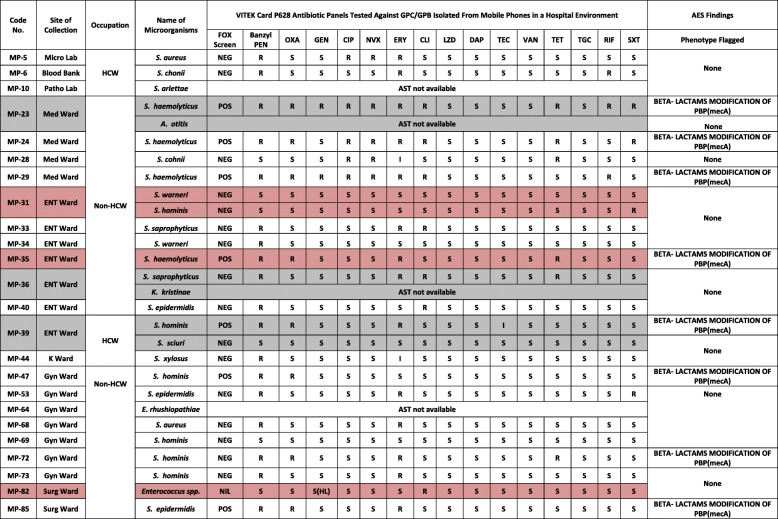
Antibiotic susceptibility and resistance pattern for GPC/GPB

*Fox Screen* cefoxitin screen, *Benzyl Pen* benzylpenicillin, *OXA* oxacillin, *GEN* gentamicin, *CIP* ciprofloxacin, *NVX* levofloxacin, *ERY* erythromycin, *CLI* clindamycin, *LZD* linezolid, *DAP* daptomycin, *TEC* teicoplanin, *VAN* vancomycin, *TET* tetracycline, *TGC* tigecycline, *RIF* rifampicin, *SXT* trimethoprim/sulfamethoxazole. *HL* high level

Gray highlight—mobile phones having growth of > 1 organisms having the same gram reaction. Pink highlight—mobile phones having mixed growth of both Gram-positive and Gram-negative organisms

*S* susceptible, *R* resistant, *POS* positive, *NEG* negative

### Statistical analysis

Data were analysed using Statistical Package for Social Sciences (SPSS) version 20. Odds ratio (OR) and confidence interval (CI) were measured using logistic regression to analyse the factors associated with a possible source of contamination of mobile phones.

## Data Availability

No datasets (culture deposits or sequence data) are generated and therefore are not applicable to this study.

## Abbreviations

AST: Antibiotic susceptibility testing
CI: Class interval
CoNS: Coagulase-negative *Staphylococci*
ENT: Ears, nose and throat
ESBL: Extended spectrum beta-lactamases
GNB: Gram-negative bacilli
GPC: Gram-positive cocci
HCW: Health care worker
K ward: Substance abuse ward
MRSA: Methicillin-resistant *Staphylococcus aureus*
Non-HCW: Non-health care worker
OR: Odds ratio
SPSS: Statistical Package for the Social Sciences
